# Association of Systolic Blood Pressure Time in Target Range With Cardiovascular Events Among PRECISION Participants

**DOI:** 10.1111/jch.70009

**Published:** 2025-07-08

**Authors:** Neel Agarwal, Julie St. John, Vikas Sunder, Ashish Sarraju, Luke J. Laffin

**Affiliations:** ^1^ Ohio State University College of Medicine Columbus Ohio USA; ^2^ Cleveland Clinic Coordinating Center for Clinical Research Cleveland Ohio USA; ^3^ Department of Cardiovascualr Medicine Section of Preventive Cardiology, Cleveland Clinic Cleveland Ohio USA

**Keywords:** blood pressure, cardiovascular outcomes, clinical trial, hypertension, time in target range

## Abstract

Blood pressure (BP) is a dynamic vital sign with variability. Novel metrics that account for BP variability and longitudinal control are gaining interest, such as time in target range (TTR) assessments. TTR is the percentage of time a patient's BP is within a desired range. We sought to determine if systolic BP TTR was associated with major adverse cardiovascular events (MACE) among participants in the PRECISION (Prospective Randomized Evaluation of Celecoxib Integrated Safety versus Ibuprofen or Naproxen) trial. PRECISION was a 24 081‐participant cardiovascular (CV) outcomes trial comparing celecoxib, naproxen, or ibuprofen in participants with increased CV risk. Systolic BP was in the target range if it was between 110 and 130 mm Hg. TTR was determined via traditional and Rosendaal linear interpolation (RLI) methods. Participants were categorized based on TTR achieved, <25%, 25%–<50%, 50%–<75%, or ≥75%. Hazard ratios (HR) and Kaplan–Meier survival curves were generated. Twenty thousand four hundred and eighty‐seven participants had at least four BP readings available for analysis and a median follow‐up of 27.6 ± 5.4 months. The cohort had a mean baseline BP of 125.2 mm Hg and a mean systolic BP of 127.5 mm Hg when accounting for all follow‐up visits. A lower risk of MACE was observed among individuals with ≥75% TTR compared with those <25% using the traditional (adjusted HR 0.70, 95% CI 0.52–0.95, *p* = 0.02) and RLI method (adjusted HR 0.56, 95% CI 0.43–0.75, *p* < 0.001). More systolic BP TTR is associated with a lower risk of MACE among individuals in PRECISION.

## 1 Introduction

Elevated blood pressure (BP) is the world's leading modifiable risk factor for attributable premature cardiovascular (CV) death [1]. Contemporary guidelines emphasize the importance of BP lowering to reduce CV risk [2, 3, 4]. Numerous analyses of prospective clinical trials and registries demonstrate that achieving a systolic BP of less than 130 mm Hg for individuals with increased CV risk leads to a reduction in major adverse cardiovascular events (MACE) [5, 6, 7, 8, 9]. Despite national guidelines and conclusive data, BP control in the United States is worsening. A recent analysis using National Health and Nutrition Examination Survey data indicated that only 43.7% of adults reported controlled hypertension, defined as the attainment of a systolic BP of under 140 mm Hg [10].

BP is a dynamic measurement with large visit‐to‐visit variability, and this variability is heightened in individuals with increased CV risk [11, 12]. Although some contemporary papers identify BP variability as an independent risk factor, modern guidelines fail to mention its impact on CV risk [2, 3, 13, 14]. Accumulating evidence suggests potential benefits to evaluating BP control by aggregating multiple values over multiple visits rather than isolated, single BP measurements [15, 16]. Methods to quantify the achievement of a target BP while incorporating BP variability are expanding and validation of these methods is needed, especially in high CV‐risk individuals [17].

Time in target range (TTR) analysis is an approach to quantify the achievement of BP goals while incorporating its variability [18]. Similar metrics, namely, BP load, which considers the percentage of BP measurement above a threshold over a period of time, demonstrate limited success outside the realm of ambulatory BP monitoring in the assessment of long‐term CV outcomes, but is useful in the diagnosis of hypertension [19, 20]. TTR, instead, considers visit‐to‐visit changes in BP to longitudinally assess BP control. A small number of post‐hoc analyses of large, specialized cohorts demonstrate that TTR may predict long‐term CV risk in patients with elevated risk of MACE [21, 22, 23, 24]. Using TTR to quantify adherence and response to treatments for hypertension and predict long‐term CV risk among general patient cohorts remains a nascent concept that has not been assessed in populations across a broad range of CV risks and comorbidities.

The PRECISION (Prospective Randomized Evaluation of Celecoxib Integrated Safety versus Ibuprofen or Naproxen) randomized clinical trial was undertaken to evaluate the CV safety of celecoxib, as compared with nonselective nonsteroidal anti‐inflammatory drugs (NSAIDs) among participants with increased CV risk. The trial gave support to the conclusion that celecoxib was noninferior to ibuprofen or naproxen in CV safety and the occurrence of MACE. Its large primary and secondary prevention patient population, with blinded CV outcome adjudication, presents an ideal cohort to assess the association of TTR with incident MACE. The present post‐hoc analysis aims to determine the association of systolic BP TTR with incident stroke, myocardial infarction (MI), and CV death in the PRECISION trial, with the hypothesis that higher systolic BP TTR is associated with a lower risk of MACE.

## 2 Methods

### 2.1 Study Population

PRECISION consisted of 24 081 participants who were randomized to receive celecoxib (*n* = 8072), naproxen (*n* = 7969), or ibuprofen (*n* = 8040). The trial enrolled participants 18 years of age and older with existing CV disease, or a high risk of future CV disease, who required NSAIDs for osteoarthritis or rheumatoid arthritis [25]. Participants had a mean follow‐up of 34.1 ± 13.4 months. Within the 30‐month window considered for this analysis, systolic BP was measured at baseline and then months 1, 2, 4, 8, 12, 18, 24, and 30.

Baseline characteristics, including patient‐reported demographics, patient‐reported comorbidities, and laboratory values, were collected. The trial's primary outcome was a composite of CV death, MI, or stroke and was adjudicated by an independent committee of treatment‐blinded specialists at the Cleveland Clinic Coordinating Center for Clinical Research (C5Research.) The first incidence of the composite of these three outcomes was used for this analysis. The data underlying this article will be shared on reasonable request to the corresponding author.

### 2.2 Clinical Characteristics and Outcomes

Systolic BP was considered within the target range if it was between 110 and 130 mm Hg, consistent with prior TTR analyses [26]. Participants were grouped based on the percentage of TTR, specifically <25%, 25%–<50%, 50%–<75%, and ≥75%.

### 2.3 BP Measurement

At each study visit, BP was collected in triplicate, with a gap of 3 min between measurements using an automated oscillometric device. Participants were asked to refrain from smoking or consuming caffeine, and a period of 5 min after rest was ensured to collect BP measurements. BP collection commenced once the participant was seated with back support and their bare arm resting at heart level. The same arm was used for each BP measurement unless medical necessity prohibited such collection. The reported BP for the visit was the average of the second and third BP measurements.

### 2.4 Statistical Analysis

The TTR for each participant was calculated by using a traditional method, which considers the percentage of BP measurements within the target range, and by the Rosendaal linear interpolation (RLI) method, which looks at the fraction of pairwise changes in BP measurements (assumes linear) in target range and then calculates the corresponding fraction of days in target range out of the total number of days assessed [27]. BP measurements and CV events after 908 days (∼30 months) were excluded and censored, respectively.

Baseline characteristics were summarized across TTR groups (<25%, 25%–<50%, 50%–<75%, ≥75%) with mean ± standard deviation (SD) or frequency and percentage reported. TTR groups were compared using a test of trend within the traditional or RLI methods.

Cox proportional hazards regression was used to assess the association between TTR and MACE. A hazard ratio (HR) with a 95% confidence interval (CI) is reported with the <25% TTR group serving as the reference group. Baseline systolic BP and treatment group were forced into every model. The list of other covariates considered for adjustment in the multivariable models included demographics (age, sex, body mass index, race, current smoker, history of coronary artery disease, peripheral arterial disease, type 2 diabetes, hypertension, heart failure, stroke, transient ischemic attack, myocardial infarction, percutaneous coronary intervention, or coronary artery bypass grafting), baseline labs (triglycerides, LDL‐C, HDL‐C, eGFR), baseline medications (aspirin, beta‐blockers, calcium channel blockers, statins, angiotensin‐converting enzyme inhibitors, angiotensin receptor blockers, thiazide diuretics, mineralocorticoid receptor antagonists), and treatment received (celecoxib, naproxen, ibuprofen). Only significant covariates were adjusted for in the final models (footnoted below appropriate tables). Subgroup by TTR achieved interactions were tested in these final multivariable models.

Additionally, Kaplan–Meier (KM) survival curves were generated for the TTR groups using both traditional and RLI analyses. Cumulative KM estimates were compared between groups using the log‐rank test. Statistical analysis was performed using SAS version 9.4 (SAS Institute Inc, Cary, NC, USA). KM curves were generated in SigmaPlot version 11.0 (Systat Software Inc, San Jose, CA, USA). Forst plot was created in Excel (Microsoft 365, Redmond, WA, USA). All tests are two‐tailed with a 0.05 significance level.

## 3 Results

There were 20 487 participants (85%) who had at least four BP readings at four different office visits, including a baseline systolic BP reading, that were available for TTR analysis. Participants in the cohort had a median of eight BP measurements, with 84% of participants having six or more BP measurements and 41% of participants having the full nine measurements considered.

### 3.1 Baseline Data

Baseline characteristics for each cohort by traditional and RLI TTR analyses are reported in Tables 1 and 2, respectively. Overall, among the 20 487 participants analyzed, the average age was 63.2 ± 9.2 years, 64.1% were female, 74.8% were White, 13.4% were Black, and 19.7% were Hispanic/Latino ethnicity. The cohort had a mean baseline systolic BP of 125.2 ± 10.4 mm Hg and a mean systolic BP of 127.5 ± 9.4 mm Hg across all BP measurements.

**TABLE 1:** Baseline characteristics of PRECISION cohort by traditional systolic blood pressure time in target range achieved.

		Traditional TTR achieved				
	Total	0%–<25%	25%–<50%	50%–<75%	75%–100%	*p* value^a^
Frequency	20 487	3900	4681	6123	5783	
Age (years)	63.2 ± 9.2	64.7 ± 8.8	63.9 ± 9.1	63.2 ± 9.3	61.8 ± 9.2	<0.001
Female, no. (%)	13 131 (64.1)	2365 (60.6)	2978 (63.6)	3985 (65.1)	3803 (65.8)	<0.001
Body mass index	32.6 ± 7.30	33.1 ± 7.24	32.7 ± 7.47	32.5 ± 7.3	32.4 ± 7.2	<0.001
Baseline systolic BP (mm Hg)	125.2 ± 10.4	131.9 ± 10.3	127.0 ± 10.9	123.3 ± 10.1	121.4 ± 7.6	<0.001
Mean systolic BP (mm Hg)	127.5 ± 9.4	137.2 ± 10.6	129.9 ± 8.5	124.8 ± 6.8	121.9 ± 4.4	<0.001
Average TTR	52.3 ± 28.2	10.9 ± 8.8	35.8 ± 6.8	59.4 ± 7.0	86.2 ± 9.3	<0.001
Race						0.22
White, no. (%)	15 326 (74.8)	2819 (72.3)	3514 (75.1)	4698 (76.7)	4295 (74.3)	
Black, no. (%)	2755 (13.4)	609 (15.6)	668 (14.3)	789 (12.9)	689 (11.9)	
Asian, no. (%)	431 (2.1)	79 (2.0)	79 (1.7)	115 (1.9)	158 (2.7)	
Other, no. (%)	1973 (9.6)	393 (10.1)	420 (9.0)	520 (8.5)	640 (11.1)	
Ethnicity						0.46
Hispanic/Latino, no. (%)	4030 (19.7)	808 (20.7)	892 (19.1)	1095 (17.9)	1235 (21.4)	
Non‐Hispanic/Latino, no. (%)	16 453 (80.3)	3092 (79.3)	3788 (80.9)	5028 (82.1)	4545 (78.6)	
Current smoker, no. (%)	4129 (20.2)	741 (19.0)	944 (20.2)	1248 (20.4)	1196 (20.7)	0.055
Coronary artery disease, no. (%)	3567 (17.5)	676 (17.4)	818 (17.6)	1115 (18.3)	958 (16.7)	0.47
Peripheral artery disease, no. (%)	329 (1.6)	61 (1.6)	84 (1.8)	88 (1.4)	96 (1.7)	0.91
Type 2 diabetes, no. (%)	7012 (34.2)	1446 (37.1)	1652 (35.3)	2002 (32.7)	1912 (33.1)	<0.001
History of hypertension, no. (%)	16 068 (78.8)	3247 (83.5)	3771 (80.9)	4820 (79.1)	4230 (73.6)	<0.001
History of HF, no. (%)	917 (4.5)	211 (5.4)	207 (4.4)	265 (4.3)	234 (4.0)	0.003
History of stroke, no. (%)	745 (3.6)	153 (3.9)	198 (4.2)	205 (3.3)	189 (3.3)	0.014
History of TIA, no. (%)	733 (3.6)	150 (3.8)	156 (3.3)	257 (4.2)	170 (2.9)	0.11
History of MI, no. (%)	1829 (8.9)	362 (9.3)	421 (9.0)	568 (9.3)	478 (8.3)	0.12
History of PCI, no. (%)	1436 (7.0)	282 (7.2)	322 (6.9)	473 (7.7)	359 (6.2)	0.14
History of CABG, no. (%)	927 (4.5)	178 (4.6)	231 (4.9)	279 (4.6)	239 (4.1)	0.16
eGFR (mL/min/1.73 m^2^)	79.8 ± 17.7	79.0 ± 17.4	78.8 ± 17.7	79.8 ± 17.9	81.1 ± 17.6	<0.001
<60	2969 (14.5)	570 (14.6)	736 (15.7)	918 (15.0)	745 (12.9)	0.003
≥60	17 518 (85.5)	3330 (85.4)	3945 (84.3)	5205 (85.0)	5038 (87.1)	
Aspirin, no. (%)	9517 (46.5)	1826 (46.8)	2211 (47.2)	2859 (46.7)	2621 (45.3)	0.09
Beta blockers, no. (%)	6174 (30.1)	1287 (33.0)	1459 (31.2)	1875 (30.6)	1553 (26.9)	<0.001
Calcium channel blockers, no. (%)	4319 (21.1)	1004 (25.7)	1067 (22.8)	1220 (19.9)	1028 (17.8)	<0.001
Statins, no. (%)	11 164 (54.5)	2084 (53.4)	2529 (54.0)	3423 (55.9)	3128 (54.1)	0.29
ACE inhibitors and/or ARBs, no. (%)	12 216 (59.6)	2539 (65.1)	2929 (62.6)	3640 (59.4)	3108 (53.7)	<0.001
Thiazide diuretics, no. (%)	4962 (24.2)	966 (24.8)	1231 (26.3)	1478 (24.1)	1287 (22.3)	<0.001
MRAs, no. (%)	263 (1.3)	45 (1.2)	63 (1.3)	85 (1.4)	70 (1.2)	0.86
Treatment group						0.004
Celecoxib	6876 (33.6)	1193 (30.6)	1544 (33.0)	2048 (33.4)	2091 (36.2)	
Ibuprofen	6800 (33.2)	1415 (36.3)	1640 (35.0)	1959 (32.0)	1786 (30.9)	
Naproxen	6811 (33.2)	1292 (33.1)	1497 (32.0)	2116 (34.6)	1906 (33.0)	

*Note*: Values are mean ± SD or *n* (%).

Abbreviation: ACE, angiotensin converting enzyme; ARB, angiotensin II receptor blocker; BP, blood pressure; CABG, coronary artery bypass graft; HF, heart failure; TIA, transient ischemic attack; eGFR, estimated glomerular filtration rate; MI, myocardial infarction; MRA, mineralocorticoid receptor antagonist; PCI, percutaneous coronary intervention.

^a^ Test of trend.

**TABLE 2:** Baseline characteristics of PRECISION cohort by Rosendaal linear interpolation systolic blood pressure time in target range achieved.

		Rosendaal TTR achieved				
	Total	0%–<25%	25%–<50%	50%–<75%	75%–100%	*p* value^a^
Frequency	20 487	5377	4172	4768	6170	
Age (years)	63.2 ± 9.2	64.5 ± 8.9	63.9 ± 9.3	62.9 ± 9.2	62.0 ± 9.3	<0.001
Female, no. (%)	13 131 (64.1)	3302 (61.4)	2711 (65.0)	3098 (65.0)	4020 (65.2)	<0.001
Body mass index	32.6 ± 7.3	32.9 ± 7.3	32.5 ± 7.3	32.6 ± 7.3	32.5 ± 7.3	0.003
Baseline systolic BP (mm Hg)	125.2 ± 10.4	130.2 ± 9.8	125.9 ± 10.4	123.3 ± 10.3	122.0 ± 9.1	<0.001
Mean SYSTOLIC BP (mm Hg)	127.5 ± 9.4	136.2 ± 10.2	128.5 ± 8.0	124.4 ± 6.4	121.7 ± 4.3	<0.001
Average TTR	51.8 ± 32.6	8.7 ± 8.4	37.9 ± 7.2	62.8 ± 7.2	90.3 ± 8.2	<0.001
Race						<0.001
White, no. (%)	15 326 (74.8)	3857 (71.7)	3113 (74.6)	3634 (76.2)	4722 (76.5)	
Black, no. (%)	2755 (13.4)	820 (15.3)	584 (14.0)	639 (13.4)	712 (11.5)	
Asian, no. (%)	431 (2.1)	114 (2.1)	87 (2.1)	84 (1.8)	146 (2.4)	
Other, no. (%)	1973 (9.6)	586 (10.9)	388 (9.3)	410 (8.6)	589 (9.5)	
Ethnicity						<0.001
Hispanic/Latino, no. (%)	4030 (19.7)	1172 (21.8)	821 (19.7)	858 (18.0)	1179 (19.1)	
Non‐Hispanic/Latino, no. (%)	16 453 (80.3)	4205 (78.2)	3350 (80.3)	3909 (82.0)	4989 (80.9)	
Current smoker, no. (%)	4129 (20.2)	1038 (19.3)	837 (20.1)	939 (19.7)	1315 (21.3)	0.014
Coronary artery disease, no. (%)	3567 (17.5)	949 (17.7)	743 (17.9)	851 (17.9)	1024 (16.7)	0.17
Peripheral artery disease, no. (%)	329 (1.6)	83 (1.5)	74 (1.8)	64 (1.3)	108 (1.8)	0.67
Type 2 diabetes, no. (%)	7012 (34.2)	1958 (36.4)	1462 (35.0)	1590 (33.3)	2002 (32.4)	<0.001
History of hypertension, no. (%)	16 068 (78.8)	4423 (82.5)	3351 (80.7)	3718 (78.3)	4576 (74.6)	<0.001
History of HF, no. (%)	917 (4.5)	269 (5.0)	195 (4.7)	195 (4.1)	258 (4.2)	0.016
History of stroke, no. (%)	745 (3.6)	211 (3.9)	154 (3.7)	169 (3.5)	211 (3.4)	0.14
History of TIA, no. (%)	733 (3.6)	206 (3.8)	145 (3.5)	180 (3.8)	202 (3.3)	0.18
History of MI, no. (%)	1829 (8.9)	508 (9.4)	398 (9.5)	425 (8.9)	498 (8.1)	0.005
History of PCI, no. (%)	1436 (7.0)	361 (6.7)	316 (7.6)	366 (7.7)	393 (6.4)	0.46
History of CABG, no. (%)	927 (4.5)	239 (4.4)	189 (4.5)	249 (5.2)	250 (4.1)	0.55
eGFR (mL/min/1.73 m^2^)	79.8 ± 17.7	79.0 ± 17.3	79.4 ± 17.9	79.9 ± 18.0	80.7 ± 17.7	<0.001
<60	2969 (14.5)	790 (14.7)	648 (15.5)	712 (14.9)	819 (13.3)	0.018
≥60	17 518 (85.5)	4587 (85.3)	3524 (84.5)	4056 (85.1)	5351 (86.7)	
Aspirin, no. (%)	9517 (46.5)	2468 (45.9)	2007 (48.1)	2190 (45.9)	2852 (46.2)	0.78
Beta blockers, no. (%)	6174 (30.1)	1740 (32.4)	1288 (30.9)	1429 (30.0)	1717 (27.8)	<0.001
Calcium channel blockers, no. (%)	4319 (21.1)	1359 (25.3)	931 (22.3)	917 (19.2)	1112 (18.0)	<0.001
Statins, no. (%)	11 164 (54.5)	2834 (52.7)	2294 (55.0)	2649 (55.6)	3387 (54.9)	0.019
ACE inhibitors and/or ARBs, no. (%)	12 216 (59.6)	3445 (64.1)	2574 (61.7)	2820 (59.1)	3377 (54.7)	<0.001
Thiazide diuretics, no. (%)	4962 (24.2)	1376 (25.6)	1032 (24.7)	1192 (25.0)	1362 (22.1)	<0.001
MRAs, no. (%)	263 (1.3)	61 (1.1)	64 (1.5)	59 (1.2)	79 (1.3)	0.78
Treatment group						0.007
Celecoxib	6876 (33.6)	1697 (31.6)	1363 (32.7)	1590 (33.3)	2226 (36.1)	
Ibuprofen	6800 (33.2)	1923 (35.8)	1423 (34.1)	1549 (32.5)	1905 (30.9)	
Naproxen	6811 (33.2)	1757 (32.7)	1386 (33.2)	1629 (34.2)	2039 (33.0)	

*Note*: Values are mean ± SD or *n* (%).

Abbreviation: ACE, angiotensin converting enzyme; ARB, angiotensin II receptor blocker; BP, blood pressure; CABG, coronary artery bypass graft; eGFR, estimated glomerular filtration rate; HF, heart failure; MI, myocardial infarction; MRA, mineralocorticoid receptor antagonist; PCI, percutaneous coronary intervention; TIA, transient ischemic attack.

^a^ Test of trend.

The cohort included 16 068 hypertensive patients with 90.6% receiving treatment for their hypertension. Hypertensive patients were documented to be taking beta‐blockers (33.5%), calcium channel blockers (24.1%), statins (57.5%), ACE inhibitors and/or angiotensin receptor blockers (67.0%), thiazide diuretics (27.7%), and mineralocorticoid receptor antagonists (1.4%). At baseline, 17.5% of participants had coronary artery disease and 4.5% had heart failure.

### 3.2 Traditional TTR

Individuals in the ≥75% TTR were younger, with an average age of 61.8 ± 9.2 years compared to the <25% TTR with an average age of 64.7 ± 8.8 years as seen in Table 1. There was a higher prevalence of type 2 diabetes (37.1% vs. 33.1%), history of hypertension (83.5% vs. 73.6%), heart failure (5.4% vs. 4.0%), transient ischemic attack (3.8% vs. 2.9%), percutaneous coronary intervention (7.2% vs. 6.2%) and the usage of beta‐blockers (33.0% vs. 26.9%), calcium channel blockers (25.7% vs. 17.8%), ACE inhibitors and/or angiotensin receptor blockers (65.1% vs. 53.7%), and thiazide diuretics (24.8% vs. 22.3%) in the <25% TTR group compared to the ≥75% TTR group (Table 1). For additional information regarding concomitant hypertensive drug usage, see Table S1A.

### 3.3 RLI TTR

Individuals with ≥75% TTR were younger, with an average age of 62.0 ± 9.3 years compared to the <25% TTR with an average age of 64.5 ± 8.9 years as seen in Table 2. For the <25% TTR group compared to the ≥75% TTR group, there was a significantly lower prevalence of smoking (19.3% vs. 21.3%) and usage of statins (52.7% vs. 54.9%), higher prevalence of type 2 diabetes (36.4% vs. 32.4%), history of hypertension (82.5% vs. 74.6%), heart failure (5.0% vs. 4.2%), MI (9.4% vs. 8.1%), and the usage of beta‐blockers (32.4% vs. 27.8%), calcium channel blockers (25.3% vs. 18.0%), ACE inhibitors and/or angiotensin receptor blockers (64.1% vs. 54.7%), and thiazide diuretics (25.6% vs. 22.1%) (Table 2). For additional information regarding concomitant hypertensive drug usage, see Table S1B.

### 3.4 Outcomes

**FIGURE 1:**
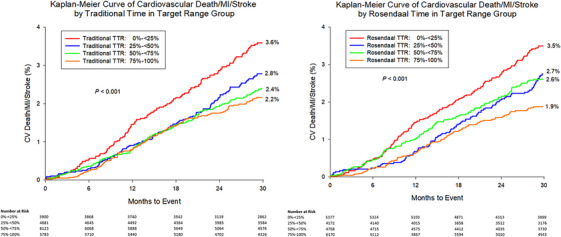
Major adverse cardiovascular event survival curves by systolic blood pressure time in target range achieved. Survival of the PRECISION trial participants, including the number at risk, stratified by time in target range achieved (TTR). (A) Traditional TTR method. (B) Rosendaal linear interpolation TTR method. Both panels demonstrate that less time in target range with respect to systolic blood pressure is associated with a higher incidence of stroke, myocardial infarction or cardiovascular death. Cumulative Kaplan–Meier estimates are given at ∼30 months for TTR group. The number at risk at each 6‐month increment is displayed below each panel. Abbreviations: CV, cardiovascular; MI, myocardial infarction; TTR, time in target range; RLI, Rosendaal linear interpolation.

The composite MACE endpoint of CV death, MI, or stroke occurred in a total of 497 participants (2.4%) (Tables S2A,B). Kaplan–Meier estimates of MACE incidence for traditional and RLI TTR analysis are shown in Figure 1. After 1 year, the incidence of CV death, MI, or stroke was 0.8% for the ≥75% traditional TTR group and 1.4% for the <25% traditional TTR group (*p* = 0.003). For RLI TTR analysis, incidence of MACE followed a similar trend (0.6% vs. 1.4%, *p* < 0.001).

Overall MACE occurrence was significantly lower among patients with ≥75% TTR (2.2%), compared to <25% TTR (3.6%), by traditional analysis (*p* < 0.001) (Figure 1). Incidence of MACE was similarly lower by RLI methodology (1.9% vs. 3.5%, *p* < 0.001) (Figure 1). These findings were corroborated by Table S2A,B for traditional and RLI TTR analysis, respectively.

Cox regression analysis was performed for both traditional and RLI TTR groups. Each TTR group was compared to the lowest achieved TTR (<25%) as demonstrated in Table 3. Using unadjusted models for both traditional and RLI analysis there was a statistically significant decrease in the risk of MACE as the percentage of TTR increased (25%–<50%, 50%–<75%, ≥75%) when compared to those in the lowest TTR group (<25%), with an HR of 0.76 (0.60–0.98, *p* = 0.034), 0.66 (0.52–0.85, *p* < 0.001), and 0.60 (0.47–0.78, *p* < 0.001) by the traditional method and an HR of 0.77 (0.60–0.98, *p* = 0.033), 0.75 (0.59–0.95, *p* = 0.015), and 0.54 (0.42–0.69, *p* < 0.001) by the RLI method.

**TABLE 3:** Association of systolic blood pressure time in target range with major adverse cardiovascular outcomes.

	Time in target range method			
	Traditional		Rosendaal linear interpolation	
	Hazard ratio (95% CI)	*p* value	Hazard ratio (95% CI)	*p* value
Unadjusted				
25%–<50%	0.76 (0.60, 0.98)	0.03	0.77 (0.60, 0.98)	0.03
50%–<75%	0.66 (0.52, 0.85)	<0.001	0.75 (0.59, 0.95)	0.02
75%–100%	0.60 (0.47, 0.78)	<0.001	0.54 (0.42, 0.69)	<0.001

Minimally adjusted				
Adjusted for baseline SBP and treatment group				
25%–<50%	0.79 (0.61, 1.02)	0.07	0.79 (0.62, 1.01)	0.06
50%–<75%	0.70 (0.55, 0.91)	0.007	0.78 (0.61, 0.99)	0.046
75%–100%	0.65 (0.49, 0.85)	0.002	0.57 (0.44, 0.73)	<0.001

Fully adjusted				
Multivariable model^a^				
25%–<50%	0.85 (0.65, 1.11)	0.24	0.81 (0.62, 1.05)	0.11
50%–<75%	0.74 (0.57, 0.98)	0.03	0.83 (0.64, 1.08)	0.17
75%–100%	0.70 (0.52, 0.95)	0.02	0.56 (0.43, 0.75)	<0.001

*Note*: Reference group above is 0%–<25% time in target range.

^a^ Adjusted for baseline systolic blood pressure, treatment, age, sex, current smoker, coronary artery disease, type 2 diabetes, history of hypertension, stroke, myocardial infarction, and baseline low density lipoprotein cholesterol.

When adjusted for only baseline systolic BP and the NSAID treatment administered in the PRECISION trial, the trend persists, with higher TTR groups (50%–<75%, ≥75%) showing a significant decrease in risk associated of incident MACE, with an HR of 0.70 (0.55–0.91, *p* = 0.007) and 0.65 (0.49–0.85, *p* = 0.002) by traditional methodology for the 50%–<75% and ≥75% ranges and an HR of 0.78 (0.61–0.99, *p* = 0.046) and 0.57 (0.44–0.73, *p* < 0.001) by RLI methodology for the 50%–<75% and ≥75%‐ranges. Finally, in the multivariable model, when fully adjusted for variables noted above, using traditional methodology, being in the 50%–<75% and ≥75% TTR groups had significantly lower risk of MACE, with an HR of 0.74 (0.57–0.98, *p* = 0.034) and 0.70 (0.52–0.95, *p* = 0.020), respectively, compared with the lowest TTR. Similarly, by the RLI method, the ≥75% TTR group had a 44% lower risk of MACE, with an HR of 0.56 (0.43–0.75, *p* < 0.001).

**FIGURE 2:**
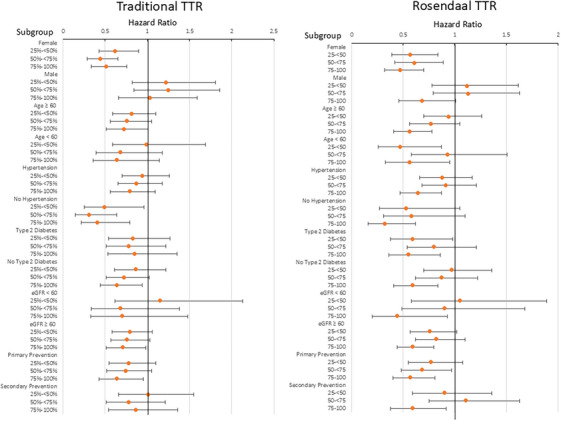
Forest plot showing the association of traditional and Rosendaal TTR achieved with major adverse cardiovascular events by subgroup for the PRECISION cohort. Hazard ratios of MACE for PRECISION trial participants, stratified by time in target range (TTR) and subgroup, and organized by (A) Traditional TTR method (B) Rosendaal linear interpolation (RLI) TTR method. Both panels exhibit similar trends with 75%–100% compliance typically exhibiting a significant decrease of associated MACE compared with the 0%–25% achieved group primarily by RLI but also by the traditional methodology.

Analysis of specific subgroups of trial participants grouped based on sex, age greater or less than 60 years, history of hypertension, history of type 2 diabetes mellitus, eGFR greater or less than 60, and primary or secondary prevention group generally demonstrated similar associations to the overall cohort—higher systolic BP TTR was associated with a lower incidence of MACE (Figure 2 and Table S3). The only subgroup by TTR achieved interaction that was significant for sex. Females showed a significant reduction in MACE for all traditional and RLI TTR groups compared to each respective 0%–25% reference group whereas associations for males were non‐significant across the board.

## 4 Discussion

This post‐hoc analysis of the PRECISION trial demonstrates that among patients with established CV disease, or an elevated CV risk, more time spent in a target systolic BP range is associated with a lower incidence of MACE, even when adjusted for baseline systolic BP and other important covariates. As a longitudinal measure, TTR summarizes the extent of which a patient's BP was within or outside a target range as a number, and as this analysis has elucidated, a unique risk factor in addition to visit BP. Across both TTR methodologies employed, traditional and RLI, there was a 30%–44% decrease in incident MACE in patients who had a BP achievement that was within 75%–100% TTR (Table 3).

Our analysis represents the largest evaluation of the association between systolic BP TTR and MACE in a well‐curated randomized clinical trial population across a wide, generalizable range of primary and secondary prevention CV risk profiles with blinded CV outcomes [28, 29]. Presently, this analysis remains the first to utilize both methodologies to assess TTR, traditional, and RLI [21, 29, 30]. Our findings are consistent with prior findings from a large European retrospective cohort that found a significantly lower risk of CV events for patients with a higher TTR [31] and from a prior 689 061 participant cohort that demonstrated an inverse and gradual association between systolic BP TTR and all‐cause mortality [32]. Other studies demonstrate similar trends in patients with specific comorbidities such as heart failure and atrial fibrillation [30, 33, 34].

Comparing the predictive value of the traditional TTR method versus the RLI TTR method has not been performed in the context of BP. It was performed in the context of systemic anticoagulation with warfarin. When the methods were compared, a traditional analysis consistently predicted a higher TTR compared to RLI [35]. As such, the authors recommended the simultaneous the presentation of both traditional and RLI methodologies to reduce bias in presentation of results, thus both were performed in the current PRECISION analysis.

Analyzing the difference between traditional and RLI TTR in the context of the subgroups analyzed provided in Table S3, yields that consistently, RLI provides significant lower hazards associated with a higher percentage TTR for those who are older or younger than 60, have a history of hypertension, have type 2 diabetes, have an eGFR lower than 60, and who are in the primary or secondary prevention group. Since RLI takes the duration of time into account and has been shown to better incorporate fluctuations in measurements than the traditional formula, there is evidence that, in these high CV risk groups, RLI may be better associated with MACE than traditional TTR [35].

Of additional merit is the significant association of traditional and RLI TTR in the female subgroup but the lack of significance in the male subgroup among PRECISION participants. One possible explanation lies with the effect androgens play in the rise of BP as males age and their late role as females enter menopause [36]. A later onset of hypertension or rise in BP in women may ultimately change their presentation of CV disease. Additionally, women may be more sensitive to adverse cardiovascular events at lower BP thresholds compared with men [37, 38].

The present analysis supports the prognostic value of systolic BP TTR for MACE. Most current quality initiatives to assess BP control often use single measurements. A prominent example is the Controlling High Blood Pressure Healthcare Effectiveness Data and Information Set (HEDIS) quality measure which is used widely within the United States to evaluate the performance of healthcare plans. However, distilling a patient's level of BP control throughout the year to a single measurement may not reflect the experience of patients, and the complexity and variability of BP trends. Systolic BP TTR should be clinically evaluated as an incrementally valuable quality metric for patients with hypertension.

Although BP fluctuations occur in all people, with increasingly aggressive systolic BP lowering recommendations in high CV‐risk patients, there may be a predisposition to hypotension, especially in older individuals [39, 40, 41]. Post‐hoc analysis of the International Verapamil‐Trandolapril Study (INVEST) found that with a systolic BP below 119 mm Hg, there was an increasing risk of MACE, similar to those with an elevated systolic BP, forming a J‐shaped risk curve [42]. By using both an upper and lower limit to define systolic BP TTR, not simply an upper limit of BP, we may also better assess CV risk.

The literature surrounding BP TTR analyses has predominantly assessed office measurements. However, recent guidelines advocate for the inclusion of ambulatory or home BP monitoring systems as a means to improve adherence to a BP control regimen among patients with hypertension and there is increasing focus on the implementation of accurate home BP monitoring technology, including wearable digital health technologies [43, 44, 45]. Whether monitoring systolic BP TTR with out‐of‐office measurements or wearable BP devices adds incremental value to patient outcomes should be explored.

## 5 Study Limitations

First, this was a non‐prespecified post‐hoc analysis. Second, BP measurements were obtained in an office/clinical setting, so the impact of white coat hypertension was not evaluated. Third, BP measurements were obtained at varied time intervals and could alter the predictive ability of the traditional approach to TTR, more so than RLI [35].

Diastolic BP was not analyzed in this study due to the lack of pre‐existing literature discussing the longitudinal control of diastolic BP within a target range. Potential future work could address the association of diastolic BP TTR with MACE, particularly given the concern that low diastolic BP may increase adverse events.

## 6 Conclusion

In this post‐hoc analysis of PRECISION trial participants, higher systolic BP TTR is associated with a lower risk of MACE among individuals with established CV disease or increased CV risk. As a longitudinal non‐invasive measure, systolic BP TTR assessment may add incremental value to CV risk prediction and BP management.

## Author Contributions

N.A., J.S., and L.L. contributed to the conception and design of the work. N.A. drafted the manuscript and figures. J.S. performed statistical analysis. J.S., A.S., V.S., and L.L. critically revised the work. L.L. supervised the work. All gave final approval and agreed to be accountable for all aspects of the work, ensuring integrity and accuracy.

## Ethics Statement

IRB approval was obtained for all sites involved with the trial.

## Consent

All patients provided written and informed consent for study participation.

## Conflicts of Interest

Luke J. Laffin has been a consultant and/or served on steering committees for Medtronic, Lilly, Mineralys Therapeutics, AstraZeneca, Idorsia, Veradermics, and Crispr Therapeutics; has received research funding from AstraZeneca; and has ownership interest in LucidAct Health and Gordy Health. The other authors have no disclosures.

## Supporting information



Supporting information

## Data Availability

The data underlying this article will be shared on reasonable request to the corresponding author.
