# Supplementary material for: Association of Systolic Blood Pressure Time in Target Range With Cardiovascular Events Among PRECISION Participants
Source: J Clin Hypertens (Greenwich). 2025 Jul 8;27(7):e70009. doi: 10.1111/jch.70009 (PMC12236988; doi:10.1111/jch.70009)
Supplement: Supplementary file 1 — Supporting information [file JCH-27-e70009-s001.docx]

Supplementary Appendix:

Table S1A. Concomitant Medications for PRECISION Cohort by Traditional Systolic Blood Pressure Time in Target Range Achieved

|  |  | Traditional TTR Achieved | | | |  |
| --- | --- | --- | --- | --- | --- | --- |
|  | **Total** | 0%-<25% | 25%-<50% | 50%-<75% | 75%-100% | *P*-value† |
| Frequency | 20,487 | 3,900 | 4,681 | 6,123 | 5,783 |  |
| Aspirin, no. (%) | 10,874 (53.1) | 2,099 (53.8) | 2,570 (54.9) | 3,266 (53.3) | 2,939 (50.8) | <0.001 |
| Beta Blockers, no. (%) | 7,791 (38.0) | 1,679 (43.1) | 1,918 (41.0) | 2,314 (37.8) | 1,880 (32.5) | <0.001 |
| Calcium Channel Blockers, no. (%) | 6,157 (30.1) | 1,544 (39.6) | 1,602 (34.2) | 1,681 (27.5) | 1,330 (23.0) | <0.001 |
| Statins, no. (%) | 13,106 (64.0) | 2,491 (63.9) | 3,006 (64.2) | 3,976 (64.9) | 3,633 (62.8) | 0.34 |
| ACE Inhibitors and/or ARBs, no. (%) | 14,066 (68.7) | 3,031 (77.7) | 3,407 (72.8) | 4,133 (67.5) | 3,495 (60.4) | <0.001 |
| Thiazide Diuretics, no. (%) | 6,478 (31.6) | 1,396 (35.8) | 1,633 (34.9) | 1,865 (30.5) | 1,584 (27.4) | <0.001 |
| MRAs, no. (%) | 525 (2.6) | 111 (2.8) | 134 (2.9) | 156 (2.5) | 124 (2.1) | 0.013 |
| Any Concomitant Hypertensive Medication | 17,709 (86.4) | 3,611 (92.6) | 4,167 (89.0) | 5,275 (86.2) | 4,656 (80.5) | <0.001 |

Values are n (%). Abbreviations: ACE = Angiotensin Converting Enzyme, ARB = Angiotensin II Receptor Blocker, MRA = mineralocorticoid receptor antagonist † Test of trend

Table S1B. Concomitant Medications for PRECISION Cohort by Rosendaal Linear Interpolation Systolic Blood Pressure Time in Target Range Achieved

|  |  | Rosendaal TTR Achieved | | | |  |
| --- | --- | --- | --- | --- | --- | --- |
|  | **Total** | 0%-<25% | 25%-<50% | 50%-<75% | 75%-100% | *P*-value† |
| Frequency | 20,487 | 5,377 | 4,172 | 4,768 | 6,170 |  |
| Aspirin, no. (%) | 10,874 (53.1) | 2,857 (53.1) | 2,295 (55.0) | 2,518 (52.8) | 3,204 (51.9) | 0.060 |
| Beta Blockers, no. (%) | 7,791 (38.0) | 2,257 (42.0) | 1,679 (40.2) | 1,791 (37.6) | 2,064 (33.5) | <0.001 |
| Calcium Channel Blockers, no. (%) | 6,157 (30.1) | 2,030 (37.8) | 1,395 (33.4) | 1,298 (27.2) | 1,434 (23.2) | <0.001 |
| Statins, no. (%) | 13,106 (64.0) | 3,383 (62.9) | 2,722 (65.2) | 3,086 (64.7) | 3,915 (63.5) | 0.72 |
| ACE Inhibitors and/or ARBs, no. (%) | 14,066 (68.7) | 4,088 (76.0) | 2,989 (71.6) | 3,212 (67.4) | 3,777 (61.2) | <0.001 |
| Thiazide Diuretics, no. (%) | 6,478 (31.6) | 1,918 (35.7) | 1,358 (32.6) | 1,528 (32.0) | 1,674 (27.1) | <0.001 |
| MRAs, no. (%) | 525 (2.6) | 150 (2.8) | 122 (2.9) | 116 (2.4) | 137 (2.2) | 0.022 |
| Any Concomitant Hypertensive Medication | 17,709 (86.4) | 4,893 (91.0) | 3,718 (89.1) | 4,102 (86.0) | 4,996 (81.0) | <0.001 |

Values are n (%). Abbreviations: ACE = Angiotensin Converting Enzyme, ARB = Angiotensin II Receptor Blocker, MRA = mineralocorticoid receptor antagonist† Test of trend

Table S2A. Frequency of Components of MACE for PRECISION Cohort By Traditional Systolic Blood Pressure Time in Target Range Achieved

|  |  | Traditional TTR Achieved | | | |  |
| --- | --- | --- | --- | --- | --- | --- |
|  | **Total** | 0%-<25% | 25%-<50% | 50%-<75% | 75%-100% | *P*-value† |
| Frequency | 20,487 | 3,900 | 4,681 | 6,123 | 5,783 |  |
| Cardiovascular Death/ MI/ Stroke | 497 (2.43) | 128 (3.28) | 120 (2.56) | 135 (2.20) | 114 (1.97) | <0.001 |
| Cardiovascular Death | 165 (0.81) | 40 (1.03) | 34 (0.73) | 47 (0.77) | 44 (0.76) | 0.19 |
| MI | 211 (1.03) | 47 (1.21) | 58 (1.24) | 59 (0.96) | 47 (0.81) | 0.061 |
| Non-fatal MI | 206 (1.01) | 47 (1.21) | 56 (1.20) | 58 (0.95) | 45 (0.78) | 0.038 |
| Stroke | 154 (0.75) | 55 (1.41) | 33 (0.70) | 37 (0.60) | 29 (0.50) | <0.001 |
| Non-fatal Stroke | 143 (0.70) | 50 (1.28) | 31 (0.66) | 35 (0.57) | 27 (0.47) | <0.001 |
| All-cause Mortality | 319 (1.56) | 64 (1.64) | 72 (1.54) | 94 (1.54) | 89 (1.54) | 0.74 |

Values are n (%). Abbreviations: MI = myocardial infarction

† Comparison of 0%-<25% and 75%-100% groups.

Table S2B. Frequency of Components of MACE for PRECISION Cohort By Rosendaal Linear Interpolation Systolic Blood Pressure Time in Target Range Achieved

|  |  | Rosendaal TTR Achieved | | | |  |
| --- | --- | --- | --- | --- | --- | --- |
|  | **Total** | 0%-<25% | 25%-<50% | 50%-<75% | 75%-100% | *P*-value† |
| Frequency | 20,487 | 5,377 | 4,172 | 4,768 | 6,170 |  |
| Cardiovascular Death/ MI/ Stroke | 497 (2.43) | 171 (3.18) | 104 (2.49) | 116 (2.43) | 106 (1.72) | <0.001 |
| Cardiovascular Death | 165 (0.81) | 53 (0.99) | 35 (0.84) | 39 (0.82) | 38 (0.62) | 0.026 |
| MI | 211 (1.03) | 64 (1.19) | 49 (1.17) | 51 (1.07) | 47 (0.76) | 0.019 |
| Non-fatal MI | 206 (1.01) | 63 (1.17) | 47 (1.13) | 50 (1.05) | 46 (0.75) | 0.018 |
| Stroke | 154 (0.75) | 67 (1.25) | 27 (0.65) | 33 (0.69) | 27 (0.44) | <0.001 |
| Non-fatal Stroke | 143 (0.70) | 62 (1.15) | 26 (0.62) | 30 (0.63) | 25 (0.41) | <0.001 |
| All-cause Mortality | 319 (1.56) | 86 (1.60) | 72 (1.73) | 76 (1.59) | 85 (1.38) | 0.33 |

Values are n (%). Abbreviations: MI = myocardial infarction

† Comparison of 0%-<25% and 75%-100% groups.

Table S3: Subgroup Analysis of the Association of Systolic Blood Pressure Time in Target Range with Major Adverse Cardiovascular Outcomes

|  |  | | Time in Target Range Method | | | |  |
| --- | --- | --- | --- | --- | --- | --- | --- |
|  | | **Traditional** | |  | **Rosendaal linear interpolation** | |  |
|  | **Hazard Ratio (95% CI)** | | ***P*-value** | **Interaction**  **Value** | **Hazard Ratio (95% CI)** | ***P*-value** | **Interaction**  **Value** |
|  |  | |  |  |  |  |  |
| Female (N = 11,362 of 13,131) | | | |  |  |  |  |
| 25%-<50% | 0.62 (0.43, 0.90) | | 0.011 | 0.002 | 0.57 (0.39, 0.84) | 0.004 | 0.028 |
| 50%-<75% | 0.44 (0.29, 0.65) | | < 0.001 |  | 0.61 (0.42, 0.89) | 0.010 |  |
| 75%-100% | 0.51 (0.34, 0.76) | | 0.001 |  | 0.47 (0.32, 0.70) | < 0.001 |  |
|  |  | |  |  |  |  |  |
| Male (N = 6,617 of 7,356) | | |  |  |  |  |  |
| 25%-<50% | 1.22 (0.82, 1.81) | | 0.34 |  | 1.12 (0.78, 1.62) | 0.54 |  |
| 50%-<75% | 1.25 (0.84, 1.86) | | 0.27 |  | 1.13 (0.79, 1.63) | 0.50 |  |
| 75%-100% | 1.03 (0.66, 1.59) | | 0.91 |  | 0.68 (0.46, 1.01) | 0.059 |  |
|  |  | |  |  |  |  |  |
| Age ≥ 60 (N = 11,828 of 13,471) | | | |  |  |  |  |
| 25%-<50% | 0.81 (0.59, 1.10) | | 0.18 | 0.58 | 0.94 (0.70, 1.26) | 0.67 | 0.076 |
| 50%-<75% | 0.76 (0.56, 1.05) | | 0.10 |  | 0.77 (0.56, 1.05) | 0.10 |  |
| 75%-100% | 0.72 (0.51, 1.01) | | 0.059 |  | 0.56 (0.41, 0.78) | < 0.001 |  |
|  |  | |  |  |  |  |  |
| Age < 60 (N = 6,151 of 7,016) | | | |  |  |  |  |
| 25%-<50% | 0.99 (0.59, 1.69) | | 0.98 |  | 0.47 (0.26, 0.87) | 0.016 |  |
| 50%-<75% | 0.68 (0.39, 1.18) | | 0.17 |  | 0.93 (0.58, 1.51) | 0.78 |  |
| 75%-100% | 0.64 (0.36, 1.14) | | 0.13 |  | 0.56 (0.33, 0.95) | 0.031 |  |
|  |  | |  |  |  |  |  |
| Hypertension (N = 14,139 of 16,068) | | | |  |  |  |  |
| 25%-<50% | 0.94 (0.70, 1.26) | | 0.69 | 0.12 | 0.88 (0.66, 1.17) | 0.37 | 0.33 |
| 50%-<75% | 0.87 (0.65, 1.18) | | 0.37 |  | 0.91 (0.68, 1.21) | 0.50 |  |
| 75%-100% | 0.79 (0.56, 1.09) | | 0.15 |  | 0.64 (0.47, 0.87) | 0.004 |  |
|  |  | |  |  |  |  |  |
| No Hypertension (N = 3,840 of 4,322) | | | |  |  |  |  |
| 25%-<50% | 0.49 (0.25, 0.96) | | 0.037 |  | 0.53 (0.27, 1.05) | 0.070 |  |
| 50%-<75% | 0.31 (0.15, 0.64) | | 0.001 |  | 0.58 (0.31, 1.10) | 0.094 |  |
| 75%-100% | 0.41 (0.22, 0.79) | | 0.007 |  | 0.32 (0.16, 0.62) | < 0.001 |  |
|  |  | |  |  |  |  |  |
| Type 2 Diabetes (N = 6,236 of 7,012) | | | |  |  |  |  |
| 25%-<50% | 0.83 (0.54, 1.27) | | 0.39 | 0.85 | 0.59 (0.38, 0.92) | 0.019 | 0.27 |
| 50%-<75% | 0.78 (0.51, 1.22) | | 0.28 |  | 0.80 (0.54, 1.21) | 0.29 |  |
| 75%-100% | 0.85 (0.53, 1.35) | | 0.49 |  | 0.55 (0.36, 0.86) | 0.008 |  |
|  |  | |  |  |  |  |  |
| No Type 2 Diabetes (N = 11,743 of 13,475) | | | |  |  |  |  |
| 25%-<50% | 0.86 (0.61, 1.22) | | 0.40 |  | 0.97 (0.70, 1.36) | 0.88 |  |
| 50%-<75% | 0.72 (0.51, 1.02) | | 0.065 |  | 0.87 (0.62, 1.22) | 0.43 |  |
| 75%-100% | 0.64 (0.44, 0.94) | | 0.021 |  | 0.59 (0.41, 0.84) | 0.004 |  |
|  |  | |  |  |  |  |  |
| eGFR < 60 (N = 2,633 of 2,969) | | | |  |  |  |  |
| 25%-<50% | 1.15 (0.62, 2.13) | | 0.65 | 0.39 | 1.05 (0.58, 1.89) | 0.87 | 0.48 |
| 50%-<75% | 0.68 (0.34, 1.38) | | 0.29 |  | 0.90 (0.49, 1.68) | 0.75 |  |
| 75%-100% | 0.70 (0.33, 1.48) | | 0.35 |  | 0.44 (0.20, 0.93) | 0.032 |  |
|  |  | |  |  |  |  |  |
| eGFR ≥ 60 (N = 15,346 of 17,518) | | | |  |  |  |  |
| 25%-<50% | 0.79 (0.58, 1.06) | | 0.12 |  | 0.76 (0.57, 1.02) | 0.067 |  |
| 50%-<75% | 0.76 (0.57, 1.03) | | 0.075 |  | 0.82 (0.62, 1.10) | 0.18 |  |
| 75%-100% | 0.71 (0.51, 0.98) | | 0.035 |  | 0.59 (0.44, 0.80) | < 0.001 |  |
|  |  | |  |  |  |  |  |
| Primary Prevention (N = 13,964 of 15,849) | | | |  |  |  |  |
| 25%-<50% | 0.78 (0.55, 1.10) | | 0.16 | 0.44 | 0.77 (0.55, 1.08) | 0.12 | 0.16 |
| 50%-<75% | 0.74 (0.52, 1.05) | | 0.09 |  | 0.68 (0.48, 0.97) | 0.032 |  |
| 75%-100% | 0.64 (0.43, 0.95) | | 0.025 |  | 0.57 (0.40, 0.81) | 0.002 |  |
|  |  | |  |  |  |  |  |
| Secondary Prevention (N = 4,015 of 4,638) | | | |  |  |  |  |
| 25%-<50% | 1.01 (0.66, 1.55) | | 0.96 |  | 0.90 (0.59, 1.36) | 0.61 |  |
| 50%-<75% | 0.78 (0.51, 1.21) | | 0.27 |  | 1.11 (0.75, 1.63) | 0.61 |  |
| 75%-100% | 0.86 (0.54, 1.36) | | 0.52 |  | 0.59 (0.38, 0.92) | 0.019 |  |

Reference group above is 0%-<25%. Adjusted for baseline systolic BP, treatment, age, sex, current smoker, coronary artery disease, type 2 diabetes, history of hypertension, stroke, myocardial infarction, baseline low density lipoprotein cholesterol.

Table S4: Association of 1 Standard Deviation Increase of Continuous Systolic Blood Pressure Time in Target Range For PRECISION Cohort

|  | Time in Target Range Method | | | | |
| --- | --- | --- | --- | --- | --- |
|  | **Traditional** | | | **Rosendaal linear interpolation** | |
|  | **Hazard Ratio (95% CI)** | | ***P*-value** | **Hazard Ratio (95% CI)** | ***P*-value** |
|  |  |  | |  |  |
| Cardiovascular Death/MI/Stroke | 0.81 (0.74, 0.88) | < 0.001 | | 0.79 (0.72, 0.86) | < 0.001 |
|  |  |  | |  |  |
| Cardiovascular Death | 0.89 (0.76, 1.03) | 0.12 | | 0.83 (0.71, 0.97) | 0.020 |
|  |  |  | |  |  |
| MI | 0.85 (0.75, 0.98) | 0.022 | | 0.85 (0.74, 0.97) | 0.020 |
|  |  |  | |  |  |
| Non-Fatal MI | 0.85 (0.0.74, 0.97) | 0.018 | | 0.85 (0.74, 0.98) | 0.020 |
|  |  |  | |  |  |
| Stroke | 0.65 (0.56, 0.77) | < 0.001 | | 0.64 (0.55, 0.76) | < 0.001 |
|  |  |  | |  |  |
| Non-Fatal Stroke | 0.66 (0.55, 0.78) | < 0.001 | | 0.64 (0.54, 0.76) | < 0.001 |
|  |  |  | |  |  |
| All-Cause Mortality | 0.94 (0.84, 1.05) | 0.27 | | 0.94 (0.84, 1.05) | 0.28 |

Table S5: Association of Time in Target Range with Outcomes Using a Target Range of Systolic BP <140 mmHg for PRECISION Cohort

|  |  | | |  | |  |  |  |
| --- | --- | --- | --- | --- | --- | --- | --- | --- |
|  | | **Traditional** | | | **Rosendaal linear interpolation** | | | |
| Number of Patients | | | | 20,487 | | | |  |
| 0%-<25% | 302 | | |  | 724 | | |  |
| 25%-<50% | 1,009 | | |  | 1,238 | | |  |
| 50%-<75% | 2,610 | | |  | 2,119 | | |  |
| 75%-100% | 16,566 | | |  | 16,406 | | |  |
|  |  | | |  |  | | |  |
|  | **Hazard Ratio (95% CI)** | | | ***P*-value** | **Hazard Ratio (95% CI)** | | | ***P*-value** |
| Cardiovascular Death/MI/Stroke | | | | |  | | |  |
| 25%-<50% | 1.71 (0.81, 3.62) | | 0.16 | | 0.94 (0.60, 1.48) | | | 0.78 |
| 50%-<75% | 1.11 (0.54, 2.30) | | 0.77 | | 0.63 (0.40, 0.98) | | 0.037 | |
| 75%-100% | 0.84 (0.42, 1.70) | | 0.63 | | 0.52 (0.36, 0.76) | | | < 0.001 |
|  |  | |  | |  | | |  |
| Cardiovascular Death | | |  | |  | | |  |
| 25%-<50% | 1.76 (0.40, 7.88) | | 0.45 | | 0.58 (0.23, 1.45) | | | 0.23 |
| 50%-<75% | 1.15 (0.27, 4.93) | | 0.85 | | 0.55 (0.24, 1.26) | | | 0.15 |
| 75%-100% | 1.21 (0.30, 4.89) | | 0.79 | | 0.64 (0.33, 1.26) | | | 0.20 |
|  |  | |  | |  | | |  |
| MI | | | | |  | | |  |
| 25%-<50% | 2.83 (0.66, 12.1) | | 0.15 | | 1.10 (0.53, 2.29) | | | 0.79 |
| 50%-<75% | 1.73 (0.41, 7.25) | | 0.45 | | 0.61 (0.29, 1.27) | | | 0.18 |
| 75%-100% | 1.48 (0.37, 5.95) | | 0.58 | | 0.63 (0.34, 1.17) | | | 0.14 |
|  |  | |  | |  | | |  |
| Non-fatal MI | | | | |  | | |  |
| 25%-<50% | 2.83 (0.66, 12.13) | | 0.15 | | 1.10 (0.53, 2.29) | | | 0.79 |
| 50%-<75% | 1.73 (0.41, 7.25) | | 0.45 | | 0.58 (0.27, 1.21) | | | 0.14 |
| 75%-100% | 1.43 (0.36, 5.77) | | 0.61 | | 0.62 (0.34, 1.14) | | | 0.12 |
|  |  | |  | |  | | |  |
| Stroke | | | | |  | | |  |
| 25%-<50% | 1.33 (0.45, 3.92) | | 0.61 | | 1.20 (0.59, 2.47) | | | 0.62 |
| 50%-<75% | 0.89 (0.32, 2.53) | | 0.84 | | 0.81 (0.40, 1.64) | | | 0.56 |
| 75%-100% | 0.46 (0.17, 1.26) | | 0.12 | | 0.37 (0.20, 0.69) | | | 0.001 |
|  |  | |  | |  | | |  |
| Non-fatal Stroke | | | | |  | | |  |
| 25%-<50% | 1.25 (0.42, 3.72) | | 0.69 | | 1.10 (0.53, 2.28) | | | 0.81 |
| 50%-<75% | 0.84 (0.29, 2.38) | | 0.74 | | 0.75 (0.37, 1.53) | | | 0.43 |
| 75%-100% | 0.43 (0.16, 1.16) | | 0.09 | | 0.34 (0.18, 0.64) | | | < 0.001 |
|  |  | |  | |  | | |  |
| All Cause Mortality | | | | |  | | |  |
| 25%-<50% | 1.86 (0.55, 6.29) | | 0.31 | | 0.82 (0.39, 1.71) | | | 0.58 |
| 50%-<75% | 1.65 (0.51, 5.33) | | 0.39 | | 0.83 (0.42, 1.62) | | | 0.58 |
| 75%-100% | 1.57 (0.50, 4.89) | | 0.44 | | 0.95 (0.53, 1.70) | | | 0.86 |

Reference group above is 0%-<25%.
